# Bioethics problems with extremely preterm infants (EPI) at birth

**DOI:** 10.1186/1824-7288-41-S2-A24

**Published:** 2015-09-30

**Authors:** Mario De Curtis, Fabio Natale, Lucia Dito, Viviana Cardilli

**Affiliations:** 1 Department of Pediatrics and Child Neuropsychiatry, University of Rome, “La Sapienza”, Italy

## Background

Each year, about 500,000 children are born in Italy and less than a thousand with a gestational age less than 26 weeks. In the past, they would very often be considered miscarriages or stillbirths. In recent years there has been a significant increase in their survival and the threshold of viability has gradually lowered to 22 weeks. The first bioethical issue on the health care of these infants involved the decision to start active care at birth. In Europe, about 19 scientific societies have published national guidelines on the care of infants < 26 weeks at birth.

## Materials and methods

The guidelines of 19 European countries on the resuscitation of EPI at birth were evaluated and some considerations were made taking into account the Italian situation [1,2].

## Results

The behavior of neonatologists as to when resuscitation should begin is very different in the 19 countries adhering to the guidelines. Whereas active resuscitation is carried out in 16 countries at 25 weeks, about half do so when dealing with an infant of 24 weeks. In eight countries, active resuscitation care is started on the basis of the clinical conditions. Before the 24^th^ week, active resuscitation is performed systematically in only 7 countries.

The involvement of parents in this decision largely varies from country to country.

## Discussion

The approach of neonatologists to the care of extremely premature newborns differs across countries, as it is influenced by different medical, social ethical and legal considerations. In some countries the decision on the start of treatment takes into account a “statistical” evaluation based on data of survival and incidence of short and long term severe disability. In other countries an “interventionist” approach is adopted which ensures all means of intensive care available to all live births. Both approaches have limitations. In Italy, even in case of extreme prematurity, every newborn attains the legal status of person and, as such, is fully entitled by the Constitution (Art. 3) to receive all the medical care he/she requires. Many Italian neonatologist think that the best approach is to individualize the care that should be based more on the patient's conditions rather than on gestational age. This individualized approach may minimize the risk prognostic error. Of course, in case of extreme prematurity, if the neonatologist realizes that any therapeutic effort is useless, intensive therapies that could translate into pursuit of futile treatment should of course be curtailed.
